# Implementation facilitators and barriers of stress first aid to protect mental health of frontline health care workers during the COVID-19 pandemic: a qualitative study

**DOI:** 10.1186/s12913-024-11812-4

**Published:** 2024-11-26

**Authors:** Shreya S. Huilgol, Lu Dong, Nabeel Qureshi, Kathryn Bouskill, Lisa S. Meredith, Courtney Gidengil

**Affiliations:** 1 RAND Corporation, Boston, MA USA; 2 RAND Corporation, Santa Monica, CA USA; 3 Cedars Sinai Medical Center, Los Angeles, CA USA

**Keywords:** Health care worker, Stress first aid, Mental health, Intervention, COVID-19 pandemic

## Abstract

**Background:**

The COVID-19 pandemic has taken a toll on frontline health care workers (HCWs), leading to poor mental and physical well-being. We conducted a large, cluster randomized controlled trial to implement an adapted Stress First Aid (SFA) intervention to support HCW well-being using a train-the-trainer (TTT) approach for rapid deployment in the United States and collected qualitative data through interviews to understand implementation. The goal of this study is to understand barriers and facilitators to deploying SFA using a TTT model, with particular emphasis on the acceptability, uptake, and barriers from the implementation.

**Methods:**

We conducted seven individual and seven group semi-structured qualitative interviews with 28 trainers (i.e., site champions) who delivered SFA training to their local HCWs from Spring 2021 to Winter 2022 in hospitals and health care centers within the United States. We utilized both inductive and deductive approaches to coding transcripts. All transcripts were coded in Dedoose. We used the Consolidated Framework for Implementation Research (CFIR) to rigorously assess implementation experiences.

**Results:**

Site champions highlighted leadership buy-in, protected time and incentives, and teams as implementation facilitators, while implementation barriers included unhelpful training materials and content, time constraints and scheduling difficulties, and pandemic-related factors, such as COVID-19 surges. SFA implementation processes varied: some champions had virtual SFA presentations, while others held informal discussions about SFA material in person. Champions also differed on their perceptions of SFA sustainability: some indicated it would be difficult to sustain SFA in their organization due to limited structure and time, while others stated they would continue to utilize it.

**Conclusion:**

Limited research has examined the implementation of HCW well-being interventions using a TTT approach in a changing environment. Site champions were able to implement SFA during a period of rapid and frequent change and shared several implementation facilitators and barriers related to the SFA intervention. In the future, addressing the implementation barriers proactively and prioritizing the implementation facilitators may prove to be useful for large-scale interventions implemented during disease outbreaks and pandemics.

**Supplementary Information:**

The online version contains supplementary material available at 10.1186/s12913-024-11812-4.

## Introduction

Since the onset of the COVID-19 pandemic, health care workers (HCWs) have reported higher levels of stress and burnout due to factors such as longer hours and staff shortages [1]. HCWs were also dealing with more uncertainty and death in their practice, especially those most involved with the treatment of patients with the most severe COVID-19 symptoms [2, 3]. The acute stress at the beginning of the COVID-19 pandemic evolved into a chronic stress situation with long-lasting effects on the mental and physical well-being of health care workers [4]. This sudden increase in stress levels requires a response to address the “new normal” many HCWs face [5]. Prior work during the COVID-19 pandemic points to the importance of peer and organizational support among health care workers to improve their well-being [6, 7].

Stress First Aid (SFA) is an evidence-informed practice that supports HCW mental and physical well-being using peer-based support strategies to identify stressful situations and address them proactively. SFA is a variation of Psychological First Aid (PFA), a more widely known practice used among service members and first responders to help support these groups deal with *acutely* stressful situations such as a large influx in COVID patients, placing patients on ventilators, or dealing with the increases in patient deaths due to COVID [8, 9]. A recent systematic review of PFA noted that the intervention reduced symptoms of anxiety, depression, posttraumatic stress, and distress, though there is ongoing need to build the evidence base for the intervention through more rigorous methods. SFA was first adapted for groups facing chronic stressful work situations such as firefighters, and was later adapted for health care workers experiencing *chronic* pandemic-related stress during the COVID-19 pandemic [8, 10, 11]. Like PFA, SFA utilizes a peer-based support system that trains individuals to recognize stress among their colleagues and leverage their relationship to their colleague to discuss and diffuse the stressful feelings.

To test the effectiveness of SFA, we conducted a cluster randomized controlled trial of SFA versus usual care among health centers and hospital units recruited during the COVID-19 pandemic in the United States [12, 13]. We used a train-the-trainer model to train site-identified champions on SFA and how to disseminate SFA competencies to workers within their own health center or hospital unit [14]. Each site selected a site champion who first received training from an expert in facilitator in SFA. The site champion then conducted their own training of site staff using implementation support materials provided by the project’s implementation team.

The use of site champions and train-the-trainer models are among the many evidence-based strategies to support the dissemination of evidence-informed practices to practitioners [15]. Previous studies have shown that site champions support implementation of evidence-based practices, through activities such as including coordinating on-site efforts to support implementation, educating colleagues, disseminating information about evidence-based practices, and leveraging existing resources to expand dissemination [16–18]. Key factors that dictate their success include their level of influence in their site, their level of ownership over the evidence-based practice, their physical presence at the test of change site, and their participative leadership style [19].

Like the use of site champions, the train-the-trainer model has also been shown to be effective in a variety of settings under a range of circumstances [20, 21]. In health care settings, the train-the-trainer model has been shown to be effective among HCWs in developing capability and capacities, even in virtual formats [22–25]. Barriers identified previously that may be exacerbated by COVID-19 relate to the need for social distancing, which may limit the ability to train staff members effectively [26]. To implement SFA, we tailored our train-the-trainer approach to support site champions. Sites selected site champions that would be best suited to disseminate the materials to on-the-ground HCWs (including being in the site of change and having support of the larger worker population). Trainings were supported but not dictated to the site champion, allowing them to take ownership of the training.

### Present study

We conducted a qualitative evaluation to assess implementation facilitators and barriers related to site champions and a train-the-trainer model associated with a large-scale deployment of SFA intervention during the COVID-19 pandemic. Qualitative studies are well suited to examine the implementation barriers, particularly those that depend on local site flexibility in a rapidly evolving context like the COVID-19 pandemic [27]. The goal of this analysis is to understand barriers and facilitators to deploying SFA during the COVID-19 pandemic through multiple waves, with particular emphasis on the acceptability, uptake, and lessons learned from the implementation.

## Methods

### Study design and procedure

We conducted semi-structured qualitative interviews throughout multiple waves of COVID-19 (from Delta wave in Spring 2021 to Omicron wave during Winter 2022) with site champions (*n* = 28) who served as the local trainers in this cluster randomized controlled trial (cRCT). Site champions completed a post-intervention interview about the acceptability and uptake of SFA and lessons learned from implementing the SFA intervention to frontline HCWs during the COVID-19 pandemic. We used qualitative data collected during a cRCT that tested the comparative effectiveness of SFA (versus usual care) to improve frontline HCW well-being during the COVID-19 pandemic [12, 13].

Detailed descriptions of the overall cRCT were reported elsewhere [12, 13]. Briefly, eight pairs of hospitals (*n* = 16 hospitals) and six pairs of health centers (*n* = 12 health centers) in the United States were recruited and participated in the current study in three waves. We recruited the hospitals and health centers in partnership with Vizient, Inc., a member-owned health care performance organization (for hospitals), and Clinical Directors Network (CDN), a practice-based research network and clinician membership and training organization (for health centers). Site recruitment was done to ensure geographic and academic diversity – sites were matched as pairs on size, type, geography, COVID-19 case rates (at the time of intervention) [12, 13]. To participate, each health center and hospital needed an appropriate match [12, 13]. Once sites were recruited and selected for the intervention, they were asked to identify at least one site champion for every 50 HCWs: larger sites had multiple site champions, whereas smaller sites had a single site champion. On average, training groups at health centers (all staff) were larger than at hospitals (single units), so there were more health center site champions.

Site champions were given training to implement a train-the-trainer model implementation strategy and prepare for their local implementation of SFA. The SFA training included several components. First, site champions were asked to watch four hours of videos on SFA and received an implementation handbook describing best strategies for implementation and sustainment, as well as materials for SFA training (e.g., slide decks, handouts, workbooks). Second, site champions participated in a two-hour virtual live SFA training and developed their plans for SFA local implementation. Once they completed training, site champions implemented their hour-long SFA training plan at their respective health center or hospital, followed by booster sessions over a 2-month period. Site champions also provided support as HCWs used the model in their daily work, and shared feedback with the study team about implementation of SFA at their sites, including barriers and facilitators. The total intervention lasted about 3–4 months, depending on the wave and cohort.

Vizient and CDN offered continuing medical education or continuing nursing education credit to site champions and HCWs who participated in the SFA training workshop as incentives. Site champions were eligible to receive two credits for participating in a two-hour workshop and HCWs received one credit for participating in the one-hour workshop facilitated by the site champions. Site champions also received lanyards, quick reference cards, and buttons with information about SFA for site champions and HCWs in the SFA sites to wear as an added incentive and visual reminder to participate.

Site champions were informed at the time of recruitment that they would be interviewed post-intervention and were contacted by email to schedule an interview within one month following the end of the intervention. Site champions were purposively sampled. If there was only one champion at a site, they were asked to be interviewed. If a site had multiple champions, we asked champions to nominate individuals with the most comprehensive perspective on SFA implementation. All selected site champions participated in post-intervention interviews. The study protocol was approved by RAND Corporation Human Subjects Protection Committee (HSPC).

### Procedure for site champion post-intervention interviews

We conducted 14 site champion semi-structured post-intervention interviews (seven as individual interviews and seven in a group format) at 11 different organizations (6 hospitals and 5 health centers) between March 2021 through July 2022. In total, 70 site champions participated in the SFA training. Of the site champions we interviewed (*n* = 28), 19 were from health centers and nine were from hospitals.

Qualitative interviews lasted approximately 20–30 min in length and were conducted virtually over Zoom.gov. All participants provided verbal consent before participating in the interview. Interviews included at least one moderator and one notetaker. Researchers with a master’s level degree or higher in a research-related field (LM, CG, NQ, LD, KB) facilitated the interviews, none of whom had prior relationships with participants [7]. Team members were trained to conduct the interviews: they conducted their first two interviews under the supervision of a senior researcher with extensive qualitative data collection experience. Team members then received feedback on how to use the interview protocol and conduct interviews to ensure that data was collected in a similar manner. After the debrief session, interviewers proceeded to conducted interviews independently.

The site champion post-intervention interview protocol (Table 1) was divided into four sections: (1) background and experience with COVID-19; (2) implementation of SFA; (3) experiences with SFA; and (4) sustaining SFA. The interview protocol was informed by the Consolidated Framework for Implementation Research (CFIR), a determinants framework to identify facilitators and barriers to successful implementation centered around six domains: implementation process, innovation, individuals, inner setting, implementation outcomes, and innovation outcomes [28]. Within these domains, site champions were asked about context-specific factors they felt would be needed to be adjusted to optimize the implementation of SFA; how SFA was received by HCWs; the effort, time, and resources needed to lead SFA, and instances where champions observed SFA practices among HCWs. Table 1 presents CFIR domains and sample questions asked.

**Table 1:** CFIR domains and sample questions from the site champion post-intervention interview protocol

CFIR Domains	Sample Questions
Implementation Process	· What attracted you to the role of being a Stress First Aid “champion” for your facility? · What were your impressions of the training you received for Stress First Aid? · Let’s walk through the steps you took to bring Stress First Aid to your facility following your initial training. [Have the champion describe how they implemented Stress First Aid] · Tell me about your experience with the ‘train-the-trainer’ approach where you trained your colleagues on Stress First Aid? · What are your thoughts on the level of effort and time it took to implement Stress First Aid at your facility?
Innovation	· One of the characteristics of Stress First Aid is that it can be adapted to particular workplace contexts. Were there any approaches you took to adapt Stress First Aid to your facility (or to a particular unit/team)?
Individuals	· How did leadership (including your immediate supervisor) at your facility react to Stress First Aid?
Inner Setting	· How did the infrastructure of your organization affect the implementation of the intervention? · How did you choose the teams/units where you implemented Stress First Aid?
Implementation Outcomes	· Would you advocate for the continuation of SFA? · What impact do you think that Stress First Aid has had on your organization as a whole? · Would it be useful to add a refresher training session for Stress First Aid? · What would you change about Stress First Aid? · Is there anything we did not cover today that you think would be important for us to understand what works well about Stress First Aid and what could be improved?
Innovation Outcomes	· How would you describe the impacts of Stress First Aid? · How did your colleagues react to Stress First Aid? · How well do you think Stress First Aid has improved the overall health and well-being of your fellow health care workers? · Have you noticed any changes in how other health care workers have been delivering care to patients? In what ways? · Did you notice if certain colleagues responded more positively to Stress First Aid than others/was Stress First Aid more effective for certain colleagues than others?

### Qualitative coding and data analysis

Interviews were audio-recorded with participant consent and transcribed, de-identified, and uploaded to Dedoose, a software program for qualitative coding, for analysis [29]. We developed a site champion codebook based on the questions in the interview protocol. We utilized a both inductive and deductive approach to coding transcripts. Researchers (SH, LD, NQ, KB) with health services and qualitative expertise co-coded a subset of interviews and met to discuss any potential points of disagreement. Once consistency was established, two researchers with qualitative health services research expertise (TB, SH) independently coded the remaining transcripts, meeting regularly to discuss any excerpts that were unclear and update the codebook. To ensure rigor, transparency, and reliability of qualitative coding, we calculated interrater reliability after 20% of the responses were coded with a pooled Cohen’s Kappa coefficient and a Cohen’s Kappa coefficient for each of the codes [30]. We calculated the Kappa scores manually. Coding procedures were discussed within team meetings and refined until the pooled Cohen’s Kappa coefficient and Cohen’s Kappa was > 0.80, which demonstrates a high level of agreement [30].

For thematic analysis, we utilized Butler Kisber’s two-stage analysis approach: we began with a coarse-grained approach in which we discussed broadly what was revealed and classified emerging themes [31]. The second, more fine-grained phase of the analysis consisted of identifying specific words, phrases, and ideas that represent larger themes. After completing the coding process, we stratified themes by sub-sample to examine the distribution of themes across site type and size (e.g., hospital versus health center), as well as cross-cutting themes to refine the intervention tools and guide subsequent implementation. The authors were involved in indexing the themes to CFIR constructs. Themes were then sorted into the CFIR domains and subdomains. We followed the Consolidated Criteria for Reporting Qualitative Research (COREQ) as a framework for data reporting.

## Results

**Fig. 1:**
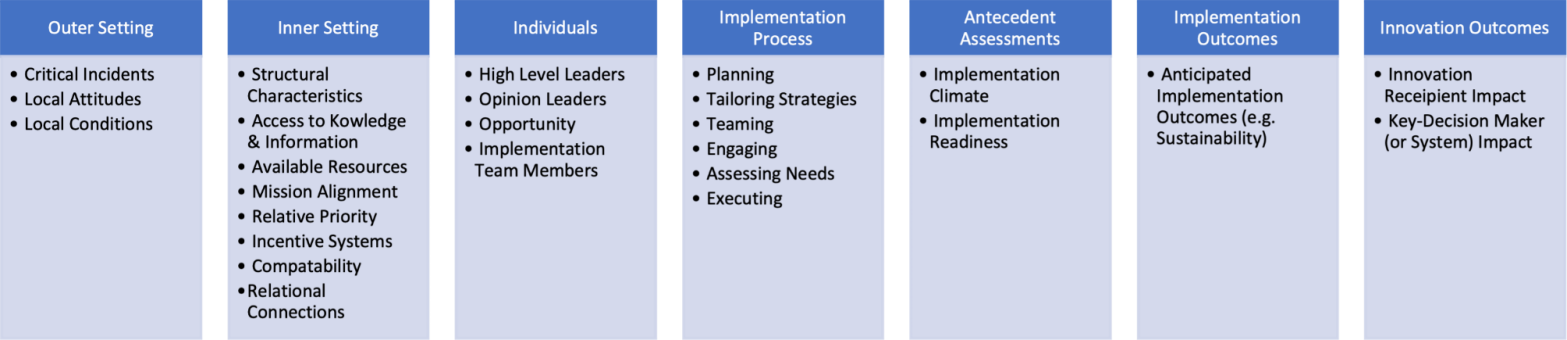
CFIR domains and constructs associated with SFA implementation themes [28]

Nineteen of the 28 champions interviewed had primarily administrative roles, seven had primarily clinical roles, and two had administrative and clinical roles. Administrative roles included site managers, directors, researchers, Chief Nursing Officers (CNOs), and educators. Of the nine champions who had any clinical role, five were physicians or other practitioners who could prescribe (i.e., physicians, physician assistants, nurse practitioners), three were behavioral health providers (i.e., licensed clinical social worker), and one was a medical assistant. Half of all site champions had protected time to prepare for their role as a site champion, with some differences by type of facility (8/19 for health centers, 6/9 for hospitals). The sites that participated in the study were geographically distributed throughout several regions of the county. Health centers were concentrated throughout the northeast and hospitals were primarily on the west coast and in the south. Our findings are organized into primary themes and subthemes and quotes are presented for each subtheme. Figure 1 provides the CFIR domains and underlying constructs, and Table 2 displays each theme and resulting CFIR constructs and domains along with an illustrative quotation.

**Table 2:** Qualitative themes associated with CFIR domains and constructs and illustrative quotations

Themes	CFIR Domain	CFIR Constructs	Illustrative Quote
SFA Implementation Facilitators			
Organizational Context (e.g., leadership buy-in)	Individuals	High-level leaders, Opinion leaders	*“I also think it was very valuable to have the leaders understanding what the training was about*,* so that I have their buy in and their support and they can come to the meetings*,* listen*,* help their caregivers understand*,* and then be able to utilize the tools in their department.”* -Site Champion, Hospital
	Inner Setting	Mission Alignment	
Practical Factors (e.g., protected time)	Inner Setting	Available Resources, Relative Priority	*“…they blocked the [HCWs] schedules off…. from 11:45 to 1:15. So we had our training from 12 to 1 until everyone was able to be there. There were no excuses like you know I have patients and stuff like that scheduled…. So it was good. I went through the training.”* -Site Champion, Health Center
Incentives	Inner Setting	Incentive Systems	*“There’s a lot of employees in the ER*,* it’s the largest department in all hospitals…Sometimes… bribery is the best way of getting friends to come and participate. So in my little welcome packet…[were] …some stickers and [a] goofy ink pen and a chill pill which was a stress ball*,* that was the shape of a pill…like I said just*,* a little bribery doesn’t hurt.”* -Site Champion, Hospital
Site Champion Teams	Inner Setting	Structural Characteristics, Relational Connections	*“So I facilitated along with [LEADERS] for the virtual setting. And I think the nice thing about that was it was split between the three of us*,* so there was a bit more comfort that like*,* if I’m forgetting something*,* I have two other co facilitators that can help me out and jump in if needed.”* -Site Champion, Health Center
	Individuals	Implementation Team Members	
	Implementation	Assessing Needs, Engaging, Teaming	
SFA Implementation Barriers			
Negative Perception of SFA Training and Materials	Inner Setting	Structural Characteristics, Available Resources, Access to Knowledge & Information	*“…. when I looked at the [SFA training] content*,* I had no idea how to try to adapt it to my setting. It seemed quite dry to me*,* and it was like*,* I have the book somewhere around here and it was like*,* do this thing*,* do this thing*,* do this thing and I tried to use some examples…it was almost academic*.” -Site Champion, Health Center
	Implementation	Planning, Tailoring Strategies	
Time-Related Constraints (e.g., time commitment to be a site champion, scheduling difficulties)	Inner Setting	Available Resources, Compatibility, Structural Characteristics, Relative Priority	*“…although we absolutely had commitment of our organization to this process*,* just by nature of the work that we do and the amount of work that we have to do and the fact that our work is right in front of us: there are patients there*,* trying to plan the timing*,* trying to make sure that this is not just another thing that the team needs to do and try to fit it in with everything else they have to do … I think was difficult… I think it may have added a little bit of stress. Stress first aid may have added a little bit of stress in some cases.”* -Site Champion, Health Center
	Individuals	Opportunity	
Pandemic-Related Factors	Outer Setting	Critical Incidents, Local Attitudes, Local Conditions	*“…we had to pause a few times because of hospital numbers going up with there being another surge throughout the course. So I think*,* just the challenges of consistency are really hard in the inpatient setting while the pandemic continues…. the effort was more so in the coordination in such a large hospital setting*,* where people have such varying demands.”* -Site Champion, Hospital
Experience and Feedback on Implementing SFA Components			
Implementation Processes Varied Widely	Inner Setting	Culture, Compatibility	*“We have a weekly all clinic meeting that is technically mandatory and is supposed to have everyone present that can be. And so I did the initial training’s in that full clinic meeting to try to get all as many residents and faculty members as possible.”* -Site Champion, Hospital *“So we went through the PowerPoint set by staff. We had different scenarios that we gave to the staff. How would they handle certain situations? And then at the end of every something like I said we would do I download it like a little relaxation yoga video with some music playing and allowed everyone to relax.”* -Site Champion, Health Center
	Implementation Process	Assessing Needs, Planning, Engaging, Executing	
	Antecedent Assessments	Implementation Climate, Implementation Readiness	
Sustainability of SFA			
Conditional with SFA Sustainability	Implementation Outcomes	Anticipated Implementation Outcomes	*“Yes [I would advocate for the continuation of SFA]*,* but [I] would want more structure and time. Because the lack of time*,* at some points*,* it felt like a burden to do that and we had to reschedule patients and find a space. If it’s more organized and we actually had [it] incorporated in our time*,* it would be actually a really good tool.”* -Site Champion, Health Center
SFA Sustainability Likely			*“Yeah I would [advocate for the continuation of SFA] and I think to make it work really well*,* I would have to do everybody in the whole hospital. Which maybe I got third of them.”* -Site Champion, Hospital
SFA Sustainability Unlikely			*“But for me… and I don’t know if there’s maybe resources available*,* but what do we do for staff now after we did our booster session and then what do we do with new staff? Like what happens? And I think that’s where I find challenging because we did all of this and okay*,* now what?”* -Site Champion, Health Center
Impact of SFA on HCW Well-Being and Organization			
Positive Impact on HCW Well-Being and Organization	Innovation Outcomes	Innovation Recipient Impact	*“…probably one of the things I like best about stress first aid is you need to watch your people that you’re working with*,* get the cues from them. Do they look stressed? Ask them if they’re okay. People liked having the permission to do that*,* you know?”* -Site Champion, Hospital
Better Quality of Patient Care	Innovation Outcomes	Innovation Recipient Impact, Key-Decision Maker (or System) Impact	“*So*,* I think when we start addressing caregiver stress and burnout*,* it automatically translates to the bedside. Caregivers are stressed*,* burnt out*,* busy*,* overwhelmed – that impacts patient care every day. We have seen an increase in our patient satisfaction scores over the last eight months in our emergency department. So*,* we’ve been watching… all the initiative we’ve been doing with staff*,* recognizing that we need to take care of our caregivers…. We’ve been watching our patient satisfaction scores increase. So*,* I would say that stress first aid was a huge impact on the care for the patients because now our staff are the better version of themselves at the patient’s bedside.”* -Site Champion, Hospital

### SFA implementation facilitators

Site champions highlighted organizational context, practical factors, and teams as implementation facilitators, most of which were within the inner setting and individual CFIR domains. First, site champions from both hospital and health center sites identified organizational context as a facilitator. For example, training site leadership or high-level leaders before rolling out SFA training to the broader organization, and having leadership buy-in and support, led to greater success with SFA implementation. Across both health centers and hospitals, site champions discussed that having clinical and administrative leadership on board with the training was important for the successful implementation of SFA because those are the people who could ensure the uptake of the SFA skills among the teams/unit they oversee. One site champion from a health center stated:

"*I think [our executive director]. is very committed to the intervention because it focused on the well-being of staff…. I mean we’re already stressed, right, because of everyday life, with the population we serve…and [our executive director] was very committed to assisting the process of implementing this intervention."*

Second, both hospital and health center site champions said that practical factors, such as providing protected time to participate as well as incentives (e.g., lunch, stickers, stress balls) to HCWs helped to increase the engagement and attendance during the SFA sessions. These factors were highly affected by factors related to the inner setting CFIR domain, such as available resources and the relative priority SFA had within the organization. Similarly, for both health centers and hospitals, site champions noted that blocking off time and making the training mandatory were effective ways to ensure HCW had time to attend sessions and not get pulled away from their work. A site champion from a hospital stated:

"*We had to make it mandatory for them to do it be able to pull them away from the work on the floor because otherwise they’re never going to have time to do it."*

If training was not mandatory and HCWs’ schedules were not cleared for SFA, champions noted that it was difficult to gather HCWs for the training.

Third, a site champion from a health center shared that deploying a team of co-facilitators or a group of site champions at one site was helpful for implementing the training. This teamwork lessened the pressure that would otherwise be on a single site champion implementing the entire SFA training at their local site. The co-facilitators then assisted as needed during the training and sessions.

### SFA implementation barriers

Site champions shared that negative perceptions of training materials, time-related constraints, and pandemic-related factors were perceived implementation barriers. First, some site champions from both hospital and health center sites reported that they needed more structure and guidance for the implementation of SFA training at their local sites. Some noted that they had difficulties providing the training to HCW (e.g., conveying the concepts and skills included in SFA to their local HCWs) and needed more instruction on how to implement the training. A site champion from a hospital stated:

*“I felt that we were kind of just handed the information and it was hard to figure out how it was going to implemented in the organization. I felt like it was a little bit unclear as to how to go over it*,* how to get everyone together on the same page so that we can all review and go over these things together.”*

There were mixed opinions on the site champion training materials, as some found the site champion training material to be too long and included materials that are not particularly relevant to their context, while others found it to be helpful. Among site champions that felt the material was not applicable either for the healthcare setting or for their particular audience, several felt that it did not achieve what they needed due to the way the training was presented and lack of guidance on implementation adoption. One site champion from a health center commented:

"...when I looked at the content, I had no idea how to try to adapt it to my setting. ...I tried to use some examples, I didn’t, it was almost academic, it was like almost like up here a little bit."

Second, for both hospital and health center sites, time constraints were notable in terms of both the scheduling difficulties for the training sessions and time consuming to complete the training. This challenge was particularly prominent when HCWs were not given protected time for SFA amid busy schedules, different shifts, and numerous responsibilities. Site champions suggested having protected time and additional training or guidance for site champions would improve SFA experiences overall. As one site champion from a health center shared:

*"So it was I think just rolling out five different trainings...was a bit...of a heavy lift, just like the coordinating of all of it even though it was like a standing meeting, it was a bit more than I anticipated."*

Third, pandemic-related factors also affected the rollout and uptake of SFA as well as HCW engagement at both hospitals and health centers. For example, there were delays in site champion training due to various COVID-19 waves (e.g., surge in cases due to the Delta variant); staff shortages; increases in staff burnout; and general lack of togetherness, both physically and virtually, due to social isolation during the pandemic. All of these factors had an impact on the implementation of SFA training at their respective local sites.

### Experience and feedback on implementing SFA Components

Hospital and health center site champions shared similar feedback on the one-hour training session. The ways site champions conducted the primary SFA training varied greatly. Some implemented virtual slide show presentations to HCW on Webex or Microsoft Teams, while others held informal discussions about SFA material in person. Certain site champions incorporated SFA into huddles or all-hands meetings at health centers, so it would be disseminated to as many HCWs as possible.

During primary implementation, other site champions indicated that their SFA sessions were conversational and a space where HCWs were able to come together and reflect on the material and past scenarios together. Some site champions from health center sites reported that they received positive feedback from HCWs, particularly when they were able to tailor the sessions towards their local sites (e.g., creating small groups, conducting virtual sessions, creating a livelier, non-work-related weekly meeting time, using informal discussions, posting materials on their internal website and encourage HCW to comment on threads).

### Sustainability of SFA

Site champions differed on their opinions regarding the sustainment of SFA. Some site champions reported that the likelihood of SFA sustainment at their local sites was conditional on having protected time for SFA sessions, incorporating prior feedback to improve the training and providing site champions with further instruction and guidance. Relatedly, other site champions indicated that they could sustain SFA using the existing tools (e.g., slide deck, workbook) provided to them as well as the perceived increase in the culture of communication because of SFA.

However, a few site champions indicated it would be difficult to sustain the use of SFA in their organization. The site champions that stated this sentiment were from the health center sites. They cited the low uptake of SFA skills among the HCW who went to the SFA training, and they were unsure about how to train new staff members to use SFA.

### Impact of SFA on HCW well-being and organization

Overall, site champions from both hospital and health center sites felt that SFA helped increase communication and provide a common language around HCW mental well-being. Site champions indicated that SFA helped HCWs recognize and manage stress and emotions, as well as increased feelings of support and cohesion in department and team culture – HCWs took more time to stop and assess both their and coworker well-being. One hospital site champion also remarked that patient care satisfaction and quality had increased after implementing SFA, because they felt that addressing caregiver stress and burnout directly translated to the bedside. As site champion shared:

*"So…these are tools that we will probably use through our entire life from now on. And we’re going to continue to use the tools that were provided, that, you know, they were very helpful."*

## Discussion

The goal of this study was to understand the facilitators and barriers site champions faced while deploying the SFA intervention during the COVID-19 pandemic, their experience and feedback implementing SFA, and the sustainability and impact of SFA on HCW well-being using the CFIR framework. Our findings indicate that facilitators for the SFA intervention heavily depended on the CFIR domains of inner setting, individuals, and implementation process. Implementation was facilitated by factors such as leadership buy-in, protected time, incentives, and site champion teams. Barriers to implementation included those same CFIR domains, as well as the domain of outer setting. The primary barriers to implementation were the negative perception of SFA training materials, time-related constraints, and pandemic-related factors. The SFA implementation process varied across sites, and site champions differed in their perceptions of SFA sustainability. Finally, site champions appeared to recognize the impact of SFA on HCW well-being and the organization because they perceived SFA as helping manage HCW mental well-being and leading to better quality of patient care.

Site champions from both hospital and health center sites broadly shared similar sentiments when asked about SFA implementation barriers and facilitators. However, one health center site champion emphasized the importance of using a team of site champions to deploy the SFA intervention to HCWs, because it lessened the burden on the individuals as the sole responsibility of implementation. A key difference across site champions from hospital and health sites was their opinions regarding SFA sustainment. Site champions from hospitals generally indicated they would advocate for the sustainment of SFA, either conditionally or using existing tools. The two site champions that stated they would not continue SFA or that it would be difficult to sustain were from health centers. We can only speculate as to possible reasons that this might have occurred. As larger and more complex organizations, hospitals may have a more developed and/or embedded approach to sustaining quality improvement or other efforts compared to health centers. Alternatively, hospital site champions may have perceived more need for SFA at their sites, and thus been more motivated to want to sustain the effort.

Based on site champions’ descriptions of implementation barriers, we propose several potential strategies to improving implementing of SFA and other train-the-trainer models [20, 32]. Site champions indicated that leadership buy-in was a prominent facilitator for implementation. Prior literature highlights the importance of leadership during the implementation process [33–36]. For example, transformational leadership has been shown to aid large-scale evidence-based practice implementation efforts, resulting in a strong and innovative climate and positive provider attitudes toward adoption [33]. For site champions, having leadership and organizational support for SFA was particularly important during an unpreceded and stressful time such as the COVID-19 pandemic. Additionally, previous studies have shown that lack of protected time is a common barrier to successful intervention delivery [37–39]. While we encouraged protected time for SFA, in addition to explicit organizational support of such efforts, ensuring protected time is critical for implementation efforts.

Another barrier to implementation that site champions noted was the negative perception of SFA training and materials. Site champions emphasized the importance of being able to adapt SFA to their local contexts. Adaptation of materials was encouraged to some extent, while recognizing that fidelity to the intervention was encouraged for the study. The negative feelings associated with these perceptions may have been exacerbated by the fact that the COVID-19 pandemic was an uncertain time with high HCW stress levels and healthcare system strain. Additionally, due to each COVID-19 wave and variant, circumstances were changing rapidly, and champions had to learn to adapt to new contexts and local conditions. Those supporting site champions moving forward should ensure an adequate balance between providing champions with enough structure for training, and explicit encouragement to tailor the training to their individual contexts.

### Implications

During the COVID-19 pandemic, HCWs across the country have faced a multitude of challenges– leading to increased stress and burnout that continues to linger [40]. SFA is an evidence-informed practice aimed at mitigating the impact of the pandemic on HCWs’ well-being, designed to address workplace stressors and facilitate peer-to-peer support. Our larger study ultimately found that health care centers had greater adherence to the intervention compared to hospitals (70% health care centers vs. 32% for hospitals), documented by the percentage of HCWs who had their attendance documented in sheets maintained by site champions [13]. Although our quantitative results indicated that the SFA intervention did not improve well-being outcomes for HCWs overall, we found the intervention had a protective effect against psychological distress and PTSD in HCWs aged 30 years or younger in health centers [13].

Little research has examined the perspectives of site champions during an intervention aimed at promoting mental well-being for HCWs during a public health crisis. A similar study implementing peer-based resilience coaching and support to health care workers during COVID-19 found similar challenges: making time with busy work schedules, balancing the uncertainty of their roles and working while experiencing burnout [41]. Through qualitative interviews, this study highlights important lessons learned and the experience of site champions with SFA implementation and resulting impact of HCW well-being. Utilizing CFIR to frame our analysis identified organizational factors that affected the uptake of SFA at health centers and hospitals. The CFIR domains of inner setting and individuals were prominent facilitators of the SFA implementation. Our findings indicate that in times of uncertainty, successful implementation relies on commitment from the entire organization. Engagement from all levels of organizational staff – not just from leadership and site champions, but also HCWs –is critical to the implementation success. Protected time for the implementation across the organization also leads to increased involvement. Although blocking off time may be difficult for organizations to implement during times of high patient volume, both site champions and HCWs appreciated the space and time for decompressing amid a stressful situation. Such an investment in HCW well-being through protected time for such activities would be valuable not only for HCWs’ own sakes, but to decrease staff turn-over and attrition. From a site champion perspective, providing a high level of support for implementation, including clear instructions with training materials, but also leaving room to adapt materials for context given constraints, is critical for successful intervention delivery.

Interventions such as SFA that are aimed at protecting HCW well-being, should be common practice within healthcare organizations, and not only deployed during times of pandemics and public health crises [7]. Since the onset of the COVID-19 pandemic, there have been a plethora of research studies focused on improving health care worker well-being: from implementing e-learning courses on resilience to peer support sessions and crisis management [36, 42–46]. The United States is facing a “Great Resignation” for health care, due to many HCWs leaving the workforce in large numbers – especially nurses [47, 48]. Staff shortages are becoming increasingly common, leading to an urgent need for HCWs. SFA and prioritizing HCW well-being may help address at workforce needs, and in turn, shortages [41, 49].

### Limitations

A primary limitation of this study relates to time sensitivity, and more specifically the variability in the timeline of SFA implementation across sites. The original study was designed to be run in three cohorts, or “waves.” Due to local conditions with COVID-19 such as the Omicron wave, cohorts were less distinct, and sites had overlapping timelines. We allowed for timeline flexibility, due to hospitals facing increased clinical demands and patient volume. Thus, we allowed hospitals to pause implementation and resume after the peak, with a targeted refresher to HCWs. This timeline may have affected some site champion SFA implementation efforts, especially towards hospitals compared to health centers during pandemic variants. We also did not collect personal demographic information for the site champions and cannot compare the characteristics of those champions who agreed to be interviewed with those who declined or could not be reached. To ensure quality and reflexivity, our study team intentionally sought to recruit a range of diverse backgrounds to lead this study to ensure that study design, data collection, analysis, and synthesis would not be biased by one particular discipline.

Additionally, we conducted both individual and group interviews with site champions. For large sites with multiple site champions, it was important to get a range of perspectives across the organization. Our goal was to understand the implementation issues across the whole unit (e.g. organization or unit), so multiple champions were necessary for larger sites. We also decided to combine individual and group interviews due to time and resource constraints. Scheduling multiple champion interviews from the same site was easier given demanding schedules and busy health care settings. This variation may have affected the results due to social pressure and external influences from peers.

## Conclusion

Our study describes site champions’ perspectives on SFA implementation, such as acceptability, likelihood of uptake, and lessons learned, and the impact on HCW well-being during the COVID-19 pandemic. Few studies have specifically examined interventions focused on improving HCW well-being using a train-the-trainer model during pandemics. The findings from this study may prove to be useful for informing future large-scale interventions implemented during disease outbreaks and pandemics.

## Supplementary Information

Supplementary Material 1.

## Data Availability

The datasets generated and/or analyzed during the current study are not publicly available due to IRB restrictions on potentially identifiable information but are available deidentified from the corresponding author on reasonable request.
