# Supplementary material for: Implementation facilitators and barriers of stress first aid to protect mental health of frontline health care workers during the COVID-19 pandemic: a qualitative study
Source: BMC Health Serv Res. 2024 Nov 26;24:1475. doi: 10.1186/s12913-024-11812-4 (PMC11600825; doi:10.1186/s12913-024-11812-4)
Supplement: Supplementary file 1 — Supplementary Material 1. [file 12913_2024_11812_MOESM1_ESM.docx]

**Full SFA Interview Protocol**

**Introduction**

1. I’d like to start by asking you about your usual role and responsibilities at your organization?
   1. How long have you been at your facility?
2. This past year has been a particularly challenging one for health care workers because of the COVID-19 pandemic. How have your experiences as a health care worker been over the course of the pandemic?
3. How would you describe your experiences as a health care worker over the course of the pandemic?
4. Prior to Stress First Aid, how did you and your colleagues cope with stress on the job? How (well) did you support one another?
5. Had your facility provided any additional support prior to implementing Stress First Aid? If so, how helpful was it?

**Implementing Stress First Aid**

1. What attracted you to the role of being a Stress First Aid “champion” for your facility?
2. What were your initial expectations of being a champion?
3. Much of Stress First Aid seems to depend upon underlying communication, teamwork, and trust among health care workers. How would you describe this at your facility? How does it differ across teams/units?
4. What were your impressions of the training you received for Stress First Aid?
5. Follow-ups: length of training, level of detail, clarity of the role, style of training?
6. Were there any aspects that you found to be unclear? Complicated?
7. Did you feel you have the support and materials you needed to prepare for the training at your local site?
8. Did you seek out any additional guidance or resources to help with training?
9. Let’s walk through the steps you took to bring Stress First Aid to your facility following your initial training. [Have the champion describe how they implemented Stress First Aid]
10. How did you relay the training back to your fellow health care workers (e.g., over a webinar, in-person)?
11. Were there any factors that you think helped facilitate Stress First Aid?
12. Were there any steps that you found particularly challenging? If so, what made them challenging?
13. How could those steps be better facilitated?
14. Were you given any protected time to participate in the SFA training? For preparing for and conducting the sessions at your site?
15. Were health care workers who attended your SFA sessions given protected time to participate?
16. How did you choose the teams/units where you implemented Stress First Aid?
17. How did you distribute information about Stress First Aid?
18. What kinds of questions about Stress First Aid did you receive?
19. Tell me about your experience with the ‘train-the-trainer’ approach where you trained your colleagues on Stress First Aid?
20. How well did it work?
21. Were there any aspects of the model that you think could be improved?
22. How did you determine the format, length, and the participants for your local training? What factors were involved in making the decisions?
23. One of the characteristics of Stress First Aid is that it can be adapted to particular workplace contexts. Were there any approaches you took to adapt Stress First Aid to your facility (or to a particular unit/team)?
24. What were these adaptations/changes?
25. How did you identify areas where adaptation may be needed?
26. What do you think this did for the intervention?
27. How did leadership (including your immediate supervisor) at your facility react to Stress First Aid?
28. What kinds of support did you receive from leadership?
29. What other kinds of support would have been useful to receive from leadership?
30. How did the infrastructure of your organization affect the implementation of the intervention?
    1. Social architecture and organization?
    2. Size of your facility? Layout?
    3. Where and how do people interact with one another these days? Is it happening virtually or in person? How do you maintain social interactions?
31. What are your thoughts on the level of effort and time it took to implement Stress First Aid at your facility?
32. Did your time and effort match what you had expected? If not, how did it differ?
33. Are there other resources that you think may have helped reduce the time and effort it took to implement Stress First Aid?

**Experiences with Stress First Aid**

1. How would you describe the impacts of Stress First Aid?
2. Can you recall a specific situation where you used the skills learned from Stress First Aid training? [Describe in detail]
3. Have you noticed your colleagues using the skills learned from Stress First Aid training (e.g., specific content from the training such as the 7Cs)?
4. How would you describe your ability to use the skills learned in Stress First Aid?
5. How about your colleagues’ ability to use the skills learned in Stress First Aid?
6. How did your colleagues react to Stress First Aid?
7. Have you tried to elicit colleagues’ reactions to Stress First Aid?
8. What kinds of feedback did you receive?
9. What aspects of Stress First Aid did your colleagues find beneficial?
10. Were there any aspects that your colleagues found complicated? Out of place? Ineffective?
11. How well do you think Stress First Aid has improved the overall health and well-being of your fellow health care workers?
12. Behavioral health?
13. Mental well-being?
14. Physical well-being?
15. Have you noticed any changes in how other health care workers have been delivering care to patients? In what ways?
16. Patient safety?
17. Patient care experiences?
18. Did you notice if certain colleagues responded more positively to Stress First Aid than others/was Stress First Aid more effective for certain colleagues than others?
19. Why do you think this was the case?
20. Did you try out any strategies to help make Stress First Aid more effective for all health care workers at your facility?
21. Did you notice any patterns with respect to who participated in the training?

**Sustaining Stress First Aid**

1. Would you advocate for the continuation of SFA?
2. If so, how would you go about advocating for Stress for Aid?
3. What kinds of support would be needed to continue Stress First Aid at your facility?
4. What impact do you think that Stress First Aid has had on your organization as a whole?
5. An increased focus on health care worker well-being?
6. Enhancement of peer support?
7. More safe discussions of safety and other interpersonal events?
8. Would it be useful to add a refresher training session for Stress First Aid?
9. What would you change about Stress First Aid?
10. Is there anything we did not cover today that you think would be important for us to understand what works well about Stress First Aid and what could be improved?
